# Community advisory board members’ perspectives on their contributions to a large multistate cluster RCT: a mixed methods study

**DOI:** 10.1017/cts.2023.673

**Published:** 2023-11-23

**Authors:** Julie Bosak, Mari-Lynn Drainoni, Mia Christopher, Bethany Medley, Sandra Rodriguez, Sydney Bell, Erin Kim, Caroline Stotz, Greer Hamilton, Carol Bigsby, Faizah Gillen, Jennifer Kimball, Craig McClay, Kim Powers, Galya Walt, Tracy Battaglia, Deborah Chassler, Linda Sprague Martinez, Karsten Lunze

**Affiliations:** 1 Boston Medical Center, Boston, MA, USA; 2 Boston University School of Public Health, Boston, MA, USA; 3 Boston University Chobanian & Avedisian School of Medicine, Boston, MA, USA; 4 Columbia University, New York, NY, USA; 5 Community Advisory Board Member, Boston, MA, USA; 6 Boston University School of Social Work, Boston, MA, USA

**Keywords:** Community-engaged research, qualitative, community advisory board, mixed methods, remote implementation, community engagement strategy

## Abstract

**Background:**

Community advisory boards (CABs) are an established approach to ensuring research reflects community priorities. This paper examines two CABs that are part of the HEALing Communities Study which aims to reduce overdose mortality. This analysis aimed to understand CAB members’ expectations, experiences, and perspectives on CAB structure, communication, facilitation, and effectiveness during the first year of an almost fully remote CAB implementation. Current literature exploring these perspectives is limited.

**Methods:**

We collected qualitative and survey data simultaneously from members (*n* = 53) of two sites’ CABs in the first 9 months of CAB development. The survey assessed trust, communication, and relations; we also conducted 32 semi-structured interviews. We analyzed the survey results descriptively. The qualitative data were analyzed using a deductive codebook based on the RE-AIM PRISM framework. Themes were drawn from the combined qualitative data and triangulated with survey results to further enrich the findings.

**Results:**

CAB members expressed strong commitment to overall study goals and valued the representation of occupational sectors. The qualitative data described a dissonance between CAB members’ commitment to the mission and unmet expectations for influencing the study within an advisory role. Survey results indicated lower satisfaction with the research teams’ ability to create a mutually beneficial process, clear communication, and sharing of power.

**Conclusion:**

Building a CAB on a remote platform, within a study utilizing a community engagement strategy, still presents challenges to fully realizing the potential of a CAB. These findings can inform more effective operationalizing of community-engaged research through enhanced CAB engagement.

## Background

Community advisory boards (CAB) are used to ensure research is designed to reflect local priorities [1–6]. The fundamental role of a CAB is to “bridge the gap between the community and researchers” and increase the community relevance of the research [7]. CABs are formed to integrate community perspectives, priorities, experiences, and knowledge into the development and conduct of the research processes, improve research quality and outcomes, and enhance dissemination [2,8–11]. The collective expertise of CABs drawn from community members representing diverse perspectives, including impacted individuals, can provide nuanced insight that inform the research process [12]. Ideally, a CAB operates as a collaborative partnership between communities and researchers, based on a foundation of power-sharing across members and a collaborative, bi-directional partnership, with an emphasis on the voice of the community [2,13].

A successful partnership between the research team and the CAB requires trusting relationships and a group environment that supports honest, transparent communication, and equity among members [13,14]. According to Gonzalez-Guarda *et al.* [13], to achieve this level of trusting collaboration, three essential conditions are needed. First, explicitly defined roles detailing expectations and relationships between entities are needed to ensure that both parties benefit from the partnership and are working toward the achievement of a shared goal. Second, the facilitator needs to prioritize power-sharing and honest communication between the two entities [13]. Lastly, capacity building among both the research team and CAB participants through trainings and Community-Based Participatory Research (CBPR) discussions enhances the likelihood of a successful collaboration and the CAB members’ ability to have a higher-level impact both within the CAB and in the wider community [2].

Although creating this type of partnership dynamic is labor intensive, it is necessary to achieve the desired goals for the researchers and the community and to realize the intended role for the CAB. This is true especially with historically marginalized populations, such as people who use drugs (PWUD) [15]. Careful attention to establishing ethical principles and guidelines for CAB functioning is crucial; this may be particularly important for research related to populations historically excluded from research such as PWUD. An effective CAB can play a central role in operationalizing aspects of CBPR including the importance of collaborative partnerships, mutual benefit, and creation of an empowering process to improve health equity [2].

As part of the pre-intervention preparation phase of the HEALing Communities Study (HCS), an implementation study using a community engagement strategy, each of the four research sites (Kentucky, Massachusetts, New York, and Ohio) was required to establish state-wide CABs. The CABs are intended to provide feedback on the HCS study design, interventions, and implementation, as well as offer local perspectives and knowledge on relevant factors that might create barriers for implementation. CAB membership and structure vary by state and include representation from members of the HCS communities, PWUD, and state agencies [16]. In two study sites, data were collected from CAB members to understand their CAB experiences. Gathering data from two state-wide CAB’s members within the same study structure presents a unique opportunity to explore many of the issues presented in the literature such as approaches to create a truly bi-directional CAB with diverse and engaged members.

Minimal literature exists exploring community participants’ perspectives on satisfaction with their individual involvement, CAB processes such as communication and group dynamic, or the overall CAB impact on a study [1,2,14]. This paper aims to enhance understanding of participants’ expectations and experiences during the HCS CABs’ first operating year with a focus on members’ perspectives on the structure, communication, facilitation, and overall effectiveness. These findings can inform future CAB creation and approaches for more effective operationalizing of CBPR to enhance the engagement of CAB members and improve their role and impact on the research.

## Methods

For this analysis, we used surveys and qualitative interviews in parallel to collect data. In analysis, we simultaneously triangulated data to compare, contrast, and integrate all findings upon completion of data collection [17].

### Study setting

The state-wide CABs are a required component of this 4-year, multi-site, parallel group, cluster-randomized waitlist-controlled trial implementing the conceptually driven Communities That HEAL intervention. Each of the four research sites used a series of questions and considerations to develop and implement their own CAB recruitment strategies. Key considerations included local stakeholders, representation among community demographics, and perspectives for decision-making. Once established, each research site worked with the CAB to create a charter including operational guidelines, decision-making rules, and communication protocols [18]. This study focuses on CABs at the two research sites that collected CAB data, Massachusetts (MA) and New York (NY). The sites had different meeting schedules, with MA having an initial in-person meeting prior to the onset of the COVID-19 pandemic followed by monthly remote sessions. NY had bi-monthly remote meetings initiated after the onset of the pandemic. Compensation schedules for CAB members were determined based on level of commitment and institutional norms for partner compensation.

In MA, CAB members were offered compensation of $200 per remote monthly meeting and in NY $100 per remote meeting.

### Survey and interview development, sampling, and collection

#### Study population

The full CAB membership in both states (MA *n* = 23 and NY *n* = 30) comprised the denominator for data collection.

#### Analytic framework

For the qualitative analysis, to maintain consistency with the HCS study, the RE-AIM/PRISM implementation science framework was utilized [19–21]. We adapted a deductive codebook and utilized the following PRISM domains: External context, Internal context, and Intervention and Implementation. The “external context” domain, which encompasses the environment in which the implementation occurs, included codes such as policy, health services environment, and COVID-19. The internal context codes covered individual CAB member characteristics and perspectives on such aspects as CAB processes and the CAB role within the study. The intervention and implementation codes included parts of the intervention such as facilitation, interaction with the research team and the community-based coalitions. The RE-AIM construct was not applicable to this stage of implementation, given its focus on outcomes. Additional codes within the three constructs were developed inductively from directed content analysis during the coding process [22].

The core components in the Community Coalition Action Theory (CCAT) provided a structured lens to interpret results and consider some of the practical components proven to impact a coalition’s functioning. CCAT postulates that breadth of representation in coalition membership influences critical processes (communication, tasks, decision-making, and cohesion) along with the impact of leadership and structure, and features how these critical processes link to participants’ satisfaction with their role and the overall coalition [23].

#### Data collection

All HCS NY and MA CAB members were invited to participate in the survey and qualitative interviews. All data collection occurred between August and November 2020 aligning with the first 9 months of implementation. We administered a 23-question survey via an email link. Questions focused on the individual’s expectations, perspective on CAB functioning, and overall CAB purpose. The survey included the 12 trust components of the validated Partnership Trust Tool. The Partnership Trust Tool uses a seven-point Likert scale ranging from 1 (very dissatisfied) to 7 (very satisfied) [24]. Participants were offered $50 for survey completion.

Researchers from the HEALing Communities Study staff conducted semi-structured individual interviews via Zoom with verbal informed consent obtained at the beginning of the interview (see Table 1 and supplemental material). Interviewees were offered $75 for participation. To protect confidentiality, a participant number was utilized to identify survey results, interview recordings, and transcripts. All data were stored in a password-protected shared drive.

**Table 1.** Excerpt of questions from the interview guide

Excerpt of interview guide questions
What do you think the role of the Community Advisory Board (CAB) is in the HEALing Communities Study (HCS)? Probe: How do you see the CAB supporting the overall study? What challenges do you see the CAB may have? Probe: Has the role of the CAB changed during the life of the HCS? What two or three things would make the HEALing Communities Study successful? (e.g. resources, policy changes, programs) In your experience so far, describe how the CAB operates (e.g. structure, leadership, communication, decision-making) How well is that working for you? Do you have suggestions for how to improve the CAB functioning? Do you foresee any challenges based on the current structure/processes?

This study protocol (Pro00038088) was approved by Advarra Inc., the HEALing Communities Study single Institutional Review Board.

### Analysis

#### Survey

Records were excluded from the analysis dataset if a participant neither consented nor completed the survey resulting in a dataset of *n* = 33. Cross-tabulation tables between state and survey items were created for all items on the survey. Each cross-tabulation was conducted as a complete-case analysis and included a frequency table displaying the counts of each response by state and percentages of each response option by state and for the total sample. For continuous items, such as age, minimum, median, mean, standard deviation, and maximum are presented by state.

#### Interviews

Cross-site consensus coding was conducted by a team of 4 coders (JB, SR, MC, BM) who developed the codebook, creating a detailed logbook as coding progressed to track adaptations. NVivo-12 software was utilized. Once reliable consensus was reached after six interviews, transcripts were individually coded with weekly meetings to discuss and resolve difficult passages. At coding completion, dominant themes were drawn across the data from both states.

#### Triangulation

Upon completion of the qualitative and quantitative data analysis, we utilized a holistic triangulation approach to align individual survey data points with qualitative themes that addressed similar content. This triangulation highlighted either supportive findings or contrasting data that allowed richer results [17]. The initial analysis was done by two of the researchers (JB and KL) and then further contextualized by the full team during a planning meeting.

## Results

In NY, 43% of participants responded to the surveys (see Table 2), and 63% participated in the interviews, with an average interview length of 31 minutes. In MA, 87% responded to the survey and a 70% participated in interviews, with an average interview time of 41 minutes. The sample included representatives from a range of occupational sectors including health care, behavioral health, public health, government officials, criminal justice, as well as PWUD and their family members [collectively identified in this paper as people with lived experience (PWLE)].

**Table 2.** Survey respondent demographics

Characteristic	MA (*n* = 20)	NY (*n* = 13)	Total (*n* = 33)
	*n* (%)	*n* (%)	*n* (%)
Ethnicity			
Hispanic or Latino/a	2 (10)	–	2 (6.1)
Race			
African American/Black	4 (20)	2 (15.4)	6 (18.2)
American Indian/Alaska Native	1 (5)	1 (7.7)	2 (6.1)
Asian	1 (5)	1 (7.7)	2 (6.1)
White	12 (60)	11 (84.6)	23 (69.7)
Other	2 (10)	–	2 (6.1)
Prefer not to answer	2 (10)	–	2 (6.1)
Gender identity			
Male	9 (45)	3 (23.1)	12 (36.4)
Female	10 (50)	10 (76.9)	20 (60.6)
Genderqueer/Gender non-conforming	1 (5)	–	1 (3)
Education			
High school degree or equivalent (e.g. GED)	2 (10)	1 (7.7)	3 (9.1)
Some colleges, no degree	4 (20)	–	4 (12.1)
Associate degree (e.g. AA, AS)	1 (5)	–	1 (3)
Bachelor’s degree (e.g. BA, BS)	7 (35)	–	7 (21.2)
Advanced degree (Masters, professional, Ph.D)	6 (30)	12 (92)	18 (55)
Age			
Mean (SD)	49.9 (11.2)	45.5 (10.7)	48.2

### Survey results

Tables 3 and 4 represent the responses regarding overall CAB experiences and components of the Partnership Trust Tool. While CAB members universally were committed to its work, they were less satisfied with their ability to influence study decisions and with communication between the CAB and the communities they represent. They ranked “good clear communication” as the most important component of trust (*n* = 32, 70%).

**Table 3.** Overall CAB experience

Survey question	Agree or agree strongly
	*N* (%)
I am committed to the work of the Consumer Advisory Board (CAB)	33 (100%)
This CAB is effective in achieving its goals	28 (84.8%)
I can influence decisions that this CAB makes	26 (78.8%)
The leaders are able to guide the CAB toward the accomplishment of its goals	24 (72.7%)
I care about the work of the CAB*	22 (68.8%)
I feel a sense of pride in what the CAB accomplishes*	19 (59.4%)

* Total *N* = 32.

**Table 4.** Research teams’ performance on components of trust

Trust component	Satisfied or very satisfied
	*N* (%)
Accessible	21 (63.6%)
Dependable	21 (63.6%)
Clear communication	14 (42.4%)
Mutually beneficial	11 (33.3%)
Openness	20 (60.6%)
Providing accurate information	22 (66.7%)
Relationship building	13 (39.4%)
Responsible*	21 (65.6%)
Sharing power and responsibility^	15 (48.4%)
Supportive	23 (69.7%)
Truthfulness*	24 (75.0%)
Value difference	21 (63.6%)

* Total *N* = 32.

Table 4 represents the satisfied and very satisfied responses (ratings of 6 or 7) of the Partnership Trust Tool.

### Qualitative results

Six themes emerged from the qualitative interview data analyzed collectively across states.

#### Theme 1: High level of commitment to the study aim of lowering overdose enhanced CAB engagement

Participants from all professional sectors expressed strong commitment to the study aim, but used slightly different language to describe the rationale for commitment. Members who identified as being in recovery or having a family member impacted by SUD framed their commitment as a personal passion.

*“When you’ve got skin in the game and your family members who’ve died, or you’ve almost died, or you’re on medication for an opioid use disorder and you’re in recovery, it becomes an important part of your life and to be able to participate at this level has been rather an honor or something.”* Behavioral Health, PWLE, CAB #11

Conversely, members who identified with a professional role described their work in more technical terminology such as mission and goals and the importance for their organization, not as a personal passion, but with a similarly strong commitment.

*“That’s the long and short of why I stick with this, because I believe in the mission…”* Health Services, Government, CAB # 30

Members referenced this strong commitment when discussing the time demands of the CAB participation such as emails and reading materials. CAB members not supported by an employer willingly volunteered their time given their desire to have an impact on implementing systems to lower overdoses.

#### Theme 2: Members from variety of sectors expressed low self-efficacy and questioned their contribution to the CAB

Some CAB members expressed hesitancy to actively speak up and participate at the larger CAB meetings, for a variety of reasons. While some expressed comfort and willingness to share their perspectives in private conversations with the facilitators, members across different sectors indicated that their reticence to speak was due to uncertainty or if their input would progress the conversation.

*“I think it’s definitely challenging for me because you have a lot of really key subject matter experts and I’m not at all a subject matter expert… I think I bring a different kind of perspective from our prevention standpoint and education awareness standpoint.”* Government, *CAB #32*

Other members expressed a lack of confidence expressing their opinions due to a basic lack of understanding of the meeting content and insufficient confidence to ask for clarification in the group setting. This lack of confidence existed within different sectors with a slightly stronger sentiment from the PWLE sector. Others at times felt compelled to express their perspective, but sensed their input was not as valued as other members. Participants did not share specific examples of having their ideas being dismissed, but the general sentiment persisted.

*“It’s not necessarily what happened, but my perception is that people that have different voices or are different stakeholders maybe are taken a little bit more seriously in their concern than I felt that I was on occasion.”* Harm Reduction, CAB #10

In contrast, others appreciated the opportunity to be included, feeling appreciative for the privilege of having a voice at the table while simultaneously questioning the value of their contribution.

*“I’m grateful to be a part of it and I’m also very happy that when I do speak, that the people do listen to me. And they’re kind, they’re courteous and they hear me, because granted, it may be merited or it may not, but at least they’re listening.”* PWLE & Health Services, *CAB # 1*

Pre-existing relationships appeared to influence members’ comfort level by participating actively in meetings. The majority of CAB members described some level of pre-existing relationship with another CAB member or connection with a member of the research team. Expressing how these relationships might impact the CAB dynamic, interviewees described feelings of increased comfort, but even with the existence of relationships some still felt hesitant about full participation.

*“I think some of us knowing each other is comforting because at least we know where each other stand on issues and we already work together, so we didn’t have to learn how to do that. And don’t necessarily have to watch what we say because we have some familiarity with each other.”* Harm Reduction, CAB # 3

#### Theme 3: Innovative and skilled facilitation diminished barriers to engagement

The CAB facilitators were perceived as expertly teasing out diverse perspectives, regardless of the individual CAB member’s personal insecurities and thoughtfully managing the presentation of information for varying levels of understanding. Members resoundingly expressed positive experiences with the facilitators.

*“I didn’t have a voice at that point … the discomfort was great, but truly it was their ability to just very carefully in such a relaxed manner explain what was going on and it helped tremendously.”* Health Services, PWLE, CAB *#6*

In addition to CAB meeting facilitation, attentive connection building between meetings increased individual members’ engagement. Interviewees described the positive impact of personalized emails and phone conversations to answer questions, elicit feedback and suggestions for upcoming meeting agendas.

Due to the onset of the COVID-19 pandemic early in the CAB formation, shifting to a teleconferencing platform required innovative facilitation skills such as creating opportunities for small group interaction via breakout rooms or adapting the meeting design. Participants appreciated the effort and adaptation to a remote platform given the large number of barriers presented during the early onset of the pandemic.

*“When they had to make the switch to virtual, I’m even more impressed with how the staff tries to make that a productive meeting, a comfortable meeting… they really try to keep the meeting going and hold our attention.”* Government, CAB # 7

A patient, inclusive style supported discussions and consensus-style decision-making that felt balanced and safe for sharing differing ideas and perspectives.

*“I think the CAB sets ground rules for participation. I think everybody is an equal player. Everybody’s perspective is respected and represented and allowed. It’s a safe environment.”* Health Services, CAB #32

#### Theme 4: Dissonance between CAB members’ expected influence versus researchers’ expectation of CAB study advisory role

Members from all sectors understood that providing expertise on areas including policy, criminal justice, full scope of addiction care and lived experience was a major goal for the CAB. Yet, members expressed a strong wish to have a more active influence on the study and the community coalitions. They felt strongly about a more tactical and operational role beyond generalized strategic advising, identifying as the “boots on the ground,” rather than removed advisors.

*“We are the Marines of the study. We’re the ones that are on the front lines, looking at what’s going on, on a day to day basis and seeing how the coalitions can work in conjunction with the study to implement.”* Criminal Justice, CAB # 12

Some CAB members were also members of their community coalitions, which gave them a direct connection to the work in the communities. Their enthusiasm and satisfaction varied based on their perception of the local coalition’s approach and functioning. Some felt their perspectives were valued by the coalition while others perceived a lack of interest from the coalitions in their CAB role.

Even with an active role in the coalition, many still felt a disconnect between their “official CAB role” and the overall influence of the CAB on the broader study. Participants expressed uncertainty around the accomplishments or any actual decision-making, and frustration about the actual impact of the CAB within the study. Minimal task-oriented processes and more of a focus on high-level concept discussion hindered a feeling of accomplishment. The exacerbation of rising overdose rates and spiraling addiction crisis during COVID-19 was repeatedly referenced as an increased cause of frustration with the lack of obvious influence and action.

At the conceptual level, CAB members articulated the goals and the high-level role of the CAB, but members in all sectors described confusion about what they personally should be doing. Frustration grew when they felt their expertise was not adequately being utilized as a “sounding board” for clear guidance.

*“I’d just like to see the meetings have more substance and just to focus on the aspects of this study that would provide real opportunities for feedback or discussion. […]* ***There [are] parts of this study that the CAB members shouldn’t have any control over.*** *But just different things that relate to the interventions going on, relate to the topic matter of overdose in Massachusetts, and just relate to what’s happening on the ground.”* Unknown sector, CAB #8

One CABs eventually created smaller subcommittees and liaison roles to each of the research cores. This created a positive influence on CAB members’ understanding of and connection to the study. These more active, task-oriented roles presented opportunities for the CAB members to directly interact with different research team members. These interactions felt positive, respectful, and impactful.

#### Theme 5: COVID-19 significantly impacted the development of CAB member relationships

Members saw their CAB participation as an opportunity to build relationships and networks. Members of differing perspectives described the opportunity to learn and broaden their understanding versus the benefit to the system of “inter-agency communication” as the purpose of the relationship. The pivot to remote meetings due to the pandemic negatively impacted these opportunities.

*“And we were beginning to build those bonds and relationships. And we’ve continued that through Zoom, but I think it’s more difficult through Zoom.”* Health Services, Government, CAB #13

Breakout rooms provided more direct contact between members, and individual introductions and “icebreaker” exercises facilitated connection between members. However, Zoom diminished ad hoc, organic conversations between members compared to in-person meetings. In addition to the few opportunities to connect with private conversations, Zoom was perceived as hindering relationship development.

*“There’s something about sitting across from somebody on a table, body language, eyes, smile or not. Someone’s sense of seeing someone’s… you can see their anger even if they’re not displaying it…You miss the in-person stuff for a variety of reasons.”* PWLE, CAB #2

For many CAB members involved in direct client services, the need to respond to COVID-19-related challenges severely limited their ability to engage in the CAB. They had to fully commit to their full-time professional roles, especially those in health care, addiction treatment, and public health. The remote aspect thus facilitated attending meetings due to the time saved from commuting to in-person meetings.

#### Theme 6: CAB members’ recognized value and challenges from the diversity of members

The diverse representation across sectors on both CABs was frequently highlighted as a positive aspect, although it presented some challenges around communication and group cohesion. Members from non-healthcare fields, or without knowledge of higher-level policy and systems felt removed from the conversation and hesitant to interject.

*“You have a very diverse group of people … everybody’s an expert in their own little area and you bring all these minds together, and I think it is so important.”* PWLE, CAB #28

Many CAB members framed their participation on the CAB as a professional networking opportunity to make cross-sector connections, learn about programming and approaches that worked in other communities across the state. They also appreciated the diversity of perspectives and many discussed their own personal growth around treatment and recovery approaches.

*“I have a more open mind than I did when this began, I mean by leaps and bounds…As long as people keep their minds open and are willing to learn from literally anyone, especially from people who may disagree, there’s something to that because that’s how minds change, for me at least.”* Health Services, PWLE, *CAB# 6*

### Mixed methods data triangulation

The holistic triangulation of survey findings and qualitative themes highlighted both alignment and some disparity between the qualitative and quantitative data (see Table 5).

**Table 5.** Triangulation of qualitative theme and related quantitative survey results

Qualitative theme	Related quantitative finding	Triangulated results
1. High level of commitment to study aim of lowering overdose enhanced engagement with the Consumer Advisory Board (CAB) role	• 100% committed to the work of the CAB	Mission-driven research evokes committed CAB members.
2. Members from variety of sectors expressed low self-efficacy and questioned their influence with participation in the CAB	• 79% felt “Ability to influence CAB decisions”	Mixed confidence and feelings about ability to influence and participate.
3. Innovative and skilled facilitation diminished barriers to engagement	• Clear communication was the top priority ranked for establishing trust • 42% believe the research team provides a “Level of Clear Communication” • 67% believe “Provides accurate information”	Successful facilitation embodies frequent, personalized interactions along with clarity of communication.
4. Dissonance between CAB members’ expected influence versus researchers’ expectation of CAB study advisory role	• 85% felt the CAB was successful in achieving its goals • 59% reported a sense of pride in what the CAB accomplishes • 48% were satisfied or very satisfied with communication with the community coalitions • 33% felt the research team promoted a mutually beneficial relationship • 48% felt positively about the research team power and responsibility sharing	Communication and power-sharing impacts members’ perception of their influence on the research and pride in the work.
5. COVID-19 significantly impacted the development of CAB member relationships	• 62% found the research team had “positive relationship building”	The remote meeting modality can impede the interactions with the broader research team.
6. CAB members’ recognized value and challenges from the diversity of members	• 69% were satisfied with the diversity of the coalition • 64% believed the research team “values differences”	The CAB embodied a respectful environment for sharing diverse perspectives.

## Discussion

This study presents findings from research data from the early stages of two CABs involved with the largest implementation study utilizing community engagement with community coalitions as the primary implementation strategy. The findings overall highlight known CAB implementation challenges and present possible constructive tactics to fully operationalize CBPR principles. The mixed methods research approach provides a rich view of CAB members’ experiences. The triangulated results identified six clear findings: (1) a mission-driven study influenced CAB members’ commitment to their role; (2) members from a variety of backgrounds expressed mixed confidence about their individual influence and fully participating in meetings; (3) frequent, personalized interactions along with clear communication are critical; (4) effective communication and power-sharing impacts members’ perception of their influence on the research and pride in the CAB work; (5) a remote meeting modality can impede interactions; (6) the CAB embodied a respectful environment for sharing diverse perspectives.

We collected data at the early stage (first year) of the CAB formation and therefore aligned our analysis with the formation stage of the CCAT. Combined, the CCAT and RE-AIM PRISM constructs added theoretical support for interpreting the findings. Due to the timing of initial CAB implementation during 2020, this study highlighted the challenges of operating through a remote platform, which placed additional pressure on role clarity, successful engagement of diverse membership, and ensuring clear, accurate communication to fully realize the benefit of a CAB and CBPR.

Members expressed lack of role clarity and varying understanding of the research advisory role in contrast with a more collaborative partnership approach. Even with up-front discussion regarding their roles, CAB members’ expectations for the CABs’ relationship with community coalitions differed from the role the research team expected from them. This surfaced especially with PWLE representatives and harm reduction staff, who might be more accustomed to an active role. Early role discussions at the initial meetings may have suffered dilution due to the onset of the COVID-19 pandemic, hiding the lack of clarity that later emerged along with subsequent frustrations. The advisory role of the CAB presents limited opportunities for specific tasks or decision-making prospects which the CCAT states can help build participant engagement and satisfaction. This finding echoes previous CAB studies, which found that satisfaction increased with CAB members’ active involvement in the full research process and financial decision-making [14]. Thus, starting with recruitment and at the initial forming of a CAB, projects may benefit from investing time collaboratively discussing and delineating a shared understanding of the CAB’s precise purpose and role within the study. Our findings provide more insight into the advantages of a mutually collaborative academic-community partnership model versus the less influential advisory role of a CAB within a study utilizing community coalitions [2,25].

The diversity of backgrounds in CAB membership seemed initially to impede collaboration. Many members expressed feelings of low self-efficacy to confidently participate early in the project due to their perceived lack of relevant knowledge or expertise. These findings are consistent with Kegler & Swan’s discoveries that diversity did not necessarily enhance functioning in the formation stage, but was realized in improved outcomes in the maintenance stage [23]. Regardless of diversity, the importance of cultivating trust for relationship building is consistently shown to be a “facilitating interpersonal factor” with coalition building [26]. Even with the diverse CAB membership and remote implementation, over half of all CAB members saw the research team as open, truthful, and valuing diversity. Prioritizing the time and energy to thoughtfully build a diverse CAB and cultivate trusting relationships is an important building block in this formation stage which requires patience and time before the benefits are realized.

The challenges of developing advisory boards through a fully remote medium became a new imperative in the midst of a global pandemic and had a strong influence on relationship building [27]. For members without pre-existing relationships and low confidence, a remote CAB created additional barriers to fully engaging with the group. Since the CAB recruitment strategy used professional networking, many participants brought a pre-existing relationship with the research team that facilitated participation and offset some barriers. The remote experience seemed to increase the need for individualized attention from either a facilitator or other research staff. This observation supports currently recommended approaches employing skilled facilitation to build individualized, in-between meeting communication and provide coaching to members on how to pursue specific contributions that highlight their area of expertise [28]. In recruiting for a remote implementation, the value of pre-existing relationships might need to be prioritized to increase level of familiarity and trust at the beginning. On the other hand, this approach might also create barriers to successfully recruiting certain target populations if the research team does not have a diverse range of pre-existing relationships. These insights for creating an effective coalition on a remote platform are especially relevant for state-wide CABs facing the barrier of broad geographic spread among participants.

The importance and challenge of communication emerged as a key finding at all different levels: between members and the facilitators, between members and the research team, and between individual members themselves. While CAB members perceived the research team as honest, they were less impressed by the quantity and style of communication. In contrast, the skilled facilitators adapted to the remote environment and successfully created individualized communication by reaching out directly to members between meetings; this did not seem to extend to the full research team. Take into consideration the context of the COVID-19 pandemic and the lack of expertise with remote platforms at that point, which likely presented practical barriers to the frequency and quality of these interactions.However, excellent facilitators recognizing the need for focused, individual conversations between meetings, increased individual members’ comfort, building trust and confidence with participation, and surfacing more diverse perspectives. The remote teleconferencing platform also hindered communication between CAB members, which required additional confidence and effort to reach out. Given the importance of communication for facilitation and for building a true partnership highlighted in prior CAB research [13,14], significant efforts should be focused on creating individualized, targeted connections between CAB members, with facilitators and the broader research staff. As evidenced in this study, the creation of opportunities for individual and small group interactions between CAB and research team members improved members’ feelings of engagement and influence [29].

### Strengths and limitations

This research presents mixed method findings on two CAB implementations. Additionally, it adds new insights of an almost fully remote implementation and the challenges of creating an effective CAB within one of the largest implementation studies utilizing a community engagement strategy in the field of addiction research conducted in the USA. To enhance the findings, we had several CAB members on the authorship team. Ongoing CAB evaluation is critical for assessing, informing, and improving CAB engagement and functioning. This research is not without limitations and only presents data from two of the four research sites. Applicable to all qualitative research is the smaller sample size, which limits the generalizability of these findings. Although the two CABs are both part of HCS, they are located in different states and have different facilitation. As such, there may be local-level nuances associated with study context, recruitment, and/or facilitation.

## Conclusion

This study’s findings add to the existing literature exploring CAB members’ perspectives on their expectations for both their individual role and the overall role of the CAB. The challenges of creating opportunities for clear influence within a large, multistate implementation study utilizing community coalitions created frustration of perceived inaction and influence for many members. The remote implementation created challenges with communication and relationship building to support group cohesion. CAB members and the study team shared a common aim, but without the physical time spent together and specific tasks to direct energy with tangible outcomes, many CAB members felt that their influence fell short of their expectation at this early stage of CAB development. These frustrations grew against the backdrop of a worsening overdose and addiction crisis due to the strains of the pandemic, highlighting the importance of ensuring PWLE have a meaningful voice within the CAB.

Insights from this research suggest approaches for building a cohesive and engaged diverse CAB on a remote platform start with a thoughtful selection of diversity of sector along with demographics that are representative of the target communities. Next, a significant up-front focus on delineating the specific role and influence of the CAB along with sharing realistic timelines for building connections and exchange of ideas. Substantial time and energy by the facilitator and the full research team must be invested in smaller, collaborative task-oriented workgroups and individualized communication to build trust, bolster engagement, and grow a truly bi-directional collaborative partnership. These findings highlight the amount of focused energy needed by both facilitators and the research team to truly operationalize CPBR principles along with strategies to ensure diverse CAB members are empowered to fully participate. Translational researchers, beyond learning the principles of a CBPR approach, must embrace the importance of CBPR, build the skillset, and commit the time, if they want to realize the benefits of a CAB on the research process.

## Supporting information

Bosak et al. supplementary material Bosak et al. supplementary material

## Supplementary material

The supplementary material for this article can be found at https://doi.org/10.1017/cts.2023.673
