# Community advisory board members’ perspectives on their contributions to a large multistate cluster RCT: a mixed methods study – CORRIGENDUM

**DOI:** 10.1017/cts.2025.58

**Published:** 2025-02-24

**Authors:** J. Bosak, M. Drainoni, M. Christopher, B. Medley, S. Rodriguez, S. Bell, E. Kim, C. Stotz, G. Hamilton, C. Bigsby, F. Gillen, J. Kimball, C. McClay, K. Powers, G. Walt, T. Battaglia, D. Chassler, L. Sprague Martinez, K. Lunze

One of the author’s names was incorrectly given in the article as originally published (Mia Christopher).

This has been corrected to Mia-Cara Christopher in the HTML and PDF versions of this article.

The authors apologise for this error.
