# Supplementary material for: Community advisory board members’ perspectives on their contributions to a large multistate cluster RCT: a mixed methods study
Source: J Clin Transl Sci. 2023 Nov 23;8(1):e1. doi: 10.1017/cts.2023.673 (PMC10879854; doi:10.1017/cts.2023.673)
Supplement: Bosak et al. supplementary material [file S2059866123006738sup001.pdf]

Community Advisory Board (CAB) Interview Guide

1. How did you become involved in the CAB?

2. What has been your experience working in CABs or other groups like this?

3. Describe your current involvement with the HCS CAB.
   Probe: In what ways have you ever worked with other members in the HCS CAB? How do you think the existing relationships impact the work of the HCS CAB?

Probe: What challenges have you experienced during your involvement with the HCS CAB? Such as in terms of time/travel commitment, in terms of representing your organization, in terms of working with other members, etc.

[MA only question] How has the transition to zoom meetings impacted the monthly CAB meetings?

4. What do you think the role of the CAB is in the HEALing Communities Study?
   Probe: How do you see the CAB supporting the overall study? What challenges do you see the CAB may have?
   Probe: Has the role of the CAB changed during the life of the HCS?

5. What two or three things would make the HEALing Communities Study successful? (e.g. resources, policy changes, programs)

6. In your experience so far, describe how the CAB operates (e.g. structure, leadership, communication, decision-making)? How well is that working for you? Do you have suggestions for how to improve the CAB functioning? Do you foresee any challenges based on the current structure/processes?

7. How can the HEALing Communities study team support the CAB?

8. What do you see as the relationship between the CAB and the community or county coalitions?

For coalition representatives: What do you see yourself bringing back to your local coalition from the CAB?

9. How representative do you think the CAB is of the stakeholders within the state? Who might be missing?
   [MA only probe] Probe: What types of changes do you foresee in your involvement in the CAB?

10. How has COVID-19 impacted the CAB?

Probe: With COVID-19 impacting the community and CAB, do you feel you have room to brainstorm and share potential solutions (i.e. learn best practices from each other, cross pollination, etc.)?

11. Do you have any other thoughts you would like to share with us about your participation in the HEALing Communities Study CAB?
